# Domestic violence-are we up to the task?

**DOI:** 10.1080/02813432.2019.1608638

**Published:** 2019-05-06

**Authors:** Emil L. Sigurdsson

**Affiliations:** Department of Family Medicine, University of Iceland, Development Center for Primary Health Care, Reykjavik, Iceland

Researches about domestic violence are neither many nor reflect the seriousness of the ailment. Despite this, some papers on that issue have been published in the Scandinavian Journal of Primary Health Care [1,2]. Being one of the leading cause of deaths by killing, this problem warrants more attention. How common is it? How are general practitioners prepared to both diagnose and deal with it? Do medical students get a adequate education about this?

By definition domestic violence is a psychological, sexual and physical abuse of one partner in relationship by the other, most commonly the male are the batterers. Sometimes referred to as intimate-partner violence (IPV) the reported prevalence is unbelievably high. In USA as much 36% of women are raped, assaulted, or stalked by intimate partners at some point during their lives [3]. In UK more than one in four women experience IPV at some time in their lives and 38% of all murdered women are murdered by their partners. In Iceland 20% of women report that they have at some time in their live been the victim of IPV. Even pregnant women are the victims of IPV, with reported prevalence as high as 16% among women attending antenatal care in the primary health care setting in Iceland [1]. Some studies even claim that IPV increases during pregnancy [4]. It is therefore extremely important that those who provide antenatal care are well awake for any signs that might indicate IPV and they may even have to screen for it. Failure to attend antenatal care should arouse suspicion and warrants further enquiry.

Women who suffer IPV, and their children, are at increased risk of several health consequences. A study from Norway has indicated that IPV could be a risk factor of pelvic inflammatory disease. Furthermore, a history of pelvic pain and gynecological surgery may be indicators of sexual abuse in childhood [2].

So how are we prepared as general practitioners to detect and assist these women? There are of course barriers for the victims to overcome to report IPV and the importance of gaining trust and encouraging these women to seek help cannot be overemphasized. A research done in UK regarding formal teaching about domestic violence and abuse in medical schools revealed that the education was not adequate [5]. Without doubt all medical school should in their medical curriculum include a thorough formal teaching on IPV, how to detect and how to support and know what resources are available to assist the victims.

Screening for IPV has not been generally accepted but Cochrane systematic review acknowledge that IPV is an important health problem and the question about screening is still debatable [6]. However, primary care providers should be alerted by certain signs that could indicate IPV. The victims of IPV can present with symptoms that are not obvious and these symptoms are often somewhat ambiguous. These signs can be dental trauma, any injury to the head and neck and defensive injuries of the forearms. Injuries to multiple areas should also raise suspicion. Chronic pain and chronic gynecologic symptoms may be a sign of abuse. Psychological symptoms such as depression, anxiety and post-traumatic disorders may also suggest IPV. Other thinks that may imply IPV are when women delay seeking treatment or are inconsistent in their explanation of injuries.

It is very important to increase education on IPV, both among medical students as well as amongst health care providers. Those who are working with accidents such a emergency rooms and fracture clinics should know when and what to look for. General practitioners, in particular, should expand their knowledge on IPV and be on the alert. Furthermore, they need to know how they and the health care and social service providers work in the best way for supporting these women with referral to the necessary channels when needed. The question about prevention is obviously very valid but a good solid evidence based proved answer is not clear. Though education and open and straightforward discussion about IPV is without doubt part of that solution. Combined effort from health authorities, communities, social workers and the police are one way of both preventing and increase and facilitate reporting of domestic violence.

Despite high prevalence numbers, IPV is without doubt still a hidden and underdiagnosed problem and neither we as general practitioner nor our communities are as well prepared as we should be.
